# Linking Tensegrity to Sports Team Collective Behaviors: Towards the Group-Tensegrity Hypothesis

**DOI:** 10.1186/s40798-020-00253-y

**Published:** 2020-06-05

**Authors:** Paulo Caldeira, Sérgio Teixeira Fonseca, Ana Paulo, Jorge Infante, Duarte Araújo

**Affiliations:** 1 Ciper, Faculdade de Motricidade Humana, Spertlab, Universidade de Lisboa, Cruz Quebrada, Dafundo Portugal; 2 Graduate Program in Rehabilitation Sciences, Universidade Federal de Minas Gerais, Belo Horizonte, Brazil; 3 Laboratoire CIAMS, Univ Paris Sud, Université Paris Sarclay, Orsay, France; 4 Laboratoire CIAMS, Université d’Orléans, Orléans, France

**Keywords:** Synergies, Tensegrity, Team Sports, Perception-Action, Informational coupling

## Abstract

Collective behaviors in sports teams emerge from the coordination between players formed from their perception of shared affordances. Recent studies based on the theoretical framework of ecological dynamics reported new analytical tools to capture collective behavior variables that describe team synergies. Here, we introduce a novel hypothesis based on the principles of tensegrity to describe collective behavior. Tensegrity principles operate in the human body at different size scales, from molecular to organism levels, in structures connected physically (biotensegrity). Thus, we propose that a group of individuals connected by information can exhibit synergies based on the same principles (group-tensegrity), and we provide an empirical example based on the dynamics of a volleyball team sub-phase of defense.

## Key Points

- Sports team collective behaviors emerge from ongoing synergies between players.
- The concept of tensegrity architecture has been related to biological organisms at multiple size scales and can be explored to further understand sports teams’ organization dynamics
- Sports teams can be regarded as group-tensegrity systems manifesting principles that allow for adaptive behavior.

## Introduction

Performance analysis of sports teams has focused on the “what,” “who,” “where,” and “when” of player behavior during competition. Notational data collection involves listing discrete actions performed in a given location on the field at specific times of the game, often relating these actions to a successful or unsuccessful outcome [1, 2]. The analysis of such data can characterize precise moments and/or discern tendencies in the game. For example, in high-level volleyball, side-out and counter-attack performances are superior when the setter is in defensive zones [3], and first tempo attacks increase the probability of scoring in transition [4]. Although notational analyses provide important discrete information, it is also important to consider the “why” and “how” of observed behaviors and their circumstances and dynamics [5]. To observe this deeper layer of events, collective variables and specific tools are already available to capture team behavior dynamics resulting from the players’ cooperative interactions to achieve common goals [6]. Research using an ecological dynamics perspective on team collective behavior [7] has identified spatial-temporal features of movement patterns both intra- and inter-teams [8–10] that reveal properties of the ongoing synergies among players.

## Sports Team Collective Behaviors

From an ecological dynamics perspective, functional group synergies occur due to processes of self-organization and coordination between players that rely on their perception of shared affordances (i.e., common possibilities for action offered by the match) [5]. During team practice, players commonly perform tasks to learn how to adjust to each other’s actions by means of perceptual attunement [11] to match affordances (i.e., become sensitized to goal-relevant sources of information). Thus, a synergy is a group action supported by match-specific information (i.e., specific circumstances) grounded in the properties of: (i) *dimensional compression*—the reduction in degrees of freedom resulting from the self-organization of the system (i.e., the team). This self-organization increases the synchrony between team elements (i.e., phase relations, see [12, 13] for details) and can be captured by collective or compound variables (e.g., cluster phase). The strength of such synchrony seems to be related to skill level and training volume [10, 12, 13]; (ii) *reciprocal compensation*—individual actions to increase performance and compensate for other less effective individual contributions [14] are associated with a player’s capacity to adapt and synchronize to others’ movements [10, 15]; (iii) *interpersonal linkages for sharing work*—interpersonal linkages occur by aggregation, interdependence, among others (see [5, 16] for a detailed discussion). The contribution of each individual to group behavior can be inferred from the area covered by the players and their distribution in the field [6], or from their movement trajectories over time [8, 13]; (iv) *degeneracy—*social networks and hypernetworks reveal the adaptability and flexibility of elements (players) as a part of a whole (team) for maintaining the desired performance. Such networks highlight the structure of organization within the team [17] and uncover the most common connection patterns [18–20], thereby identifying differences in team strategies [21, 22].

Although the quantification of these synergistic properties with these methods elucidates synergetic behavior during competitive team sports, we hypothesized that an effective organization of sports teams may also be described as tensegrity systems. Specifically, a team corresponds to a large tensegrity system made of smaller tensegrity subsystems (players) connected by previously learned information and by information available in their performance environment. The conceptualization of sports teams as tensegrity systems can complement the information provided by the measurements of the properties described above, since it provides means to capture the initial conditions of a team as well as the influence of learned and trained processes in team positional configuration.

This novel approach may help coaches and sports professionals to understand how teams maintain their integrity (structural stability) despite constant individual changes (player actions and perceptions). Before introducing this hypothesis, we present a review of the literature on the dynamical properties of tensegrity structures [23, 24] and their applications to mobile tensegrity architectures [25–27].

## Tensegrity and Biotensegrity

The term “tensegrity” was first used in 1962 by the architect Buckminster Fuller to describe structures which maintain their integrity by global tension distribution (i.e., tension may be registered at the level of the structure as a whole unit) [28, 29]. More recently, Motro (2003, in particular pp.19–23) proposed a broader definition of a tensegrity state: “A tensegrity state is a stable self-equilibrated state of a system containing a discontinuous set of compressed components inside a continuum of tensioned component” (p. 19). Other definitions of tensegrity and tensegrity systems can be found in the literature [24, 28, 30, 31]. Tensegrity encapsulates the following set of properties: (i) a pre-stress condition to reach equilibrium, which is a state of intrinsic tension allowing fast responses to changes in stress anywhere in the system; (ii) energetic efficiency, as tensegrity system configurations favor efficiency and are capable of storing energy within the system itself; (iii) nonlinear viscoelastic type properties such as non-linear stiffening, which allow tensegrity systems to become stronger when subjected to higher forces; and (iv) omnidirectional stability is the ability to maintain functional properties independently of gravity direction [32, 33]. Tensegrity structures occur in many areas, such as architecture [34], art [35], engineering [36], robotics [37], biological cell models [38], and human systems [39, 40]. Tensegrity structures have high levels of functionality with energetic efficiency due to their synergetic geometry (i.e., the geometry that underlies the mechanics) [29]. Tensegrity structures in living organisms (biotensegrity) at multiple size scales is a complex phenomenon and has increasingly received attention from researchers [41]. However, these studies have focused on single organisms, as the tensegrity structures analyzed have their components physically connected. In the present article, we explore the hypothesis that a group of individuals, such as a sports team, may also behave as a larger group-tensegrity system connected by information.

Tensegrity is a dynamic property comprising a tension network and a movement system [32]. The foundation of tensegrity structures is their geometry and geodesic and triangular organizations. Straight lines connecting the center of circles form hexagons and triangles, rendering a structure with higher strength and resilience [29]. Because geodesic geometry achieves the most efficient arrangement of space and materials, it is unsurprising that tensegrity structures are common in the natural world (e.g., viruses, proteins, carbon atoms, cells). Over the last decades, tensegrity architecture have been applied to biological organisms at multiple size scales, including molecular [42], cellular [43], tissue [44], organ [40], and organ system [39, 45] levels. Examples are the self-stabilization properties of proteins and DNA [42], interactions between cells and the extracellular matrix controlling embryo patterning [38], muscle cells regulating muscle fiber size [46], lung fiber support system [40], the human spine [47], the muscular-ligament-skeletal system [48], and the haptic perception system [39].

### Tensegrity as an Explanation for the Structural Stability of Complex Biological Systems

In biological organisms, self-assembly takes place as smaller units form larger stable structures with unique properties that were absent in the individual components, ultimately resulting in an organization of systems within systems [41]. Although the connectivity is maintained between systems, hierarchies are established, and multiple states emerge from ongoing synergies. Parts of a synergy are synergies themselves, and they are function-, task-, and context-specific [49]. This process is congruent with the behavior of biological micro tensegrity structures and with macro-level interactions between tensegrity systems, as in those occurring during complex movements in humans. Tensegrity, as Turvey and Fonseca [39] insightfully wrote, “is a good biological model for Bernstein’s level of synergies” (p. 152)*.* Bernstein’s (1967, 1996) work was fundamental to understand motor control, coordination, and the mechanisms whereby functional units combine to reduce the number of degrees of freedom for meeting task demands. To organize complex global movements, the muscular-ligament-skeletal system is “supported by the basement level of tone” ([39], p. 143), which corresponds to the pre-stress property of tensegrity structures. The architecture at the level of tonus is a multi-fractal biotensegrity system exhibiting pre-stress at all levels, which allows system stability and fast adaptation to mechanical perturbations by re-distribution of tension. This pre-stress characteristic conveys the necessary support for self-organizing processes that enables synergies [39]. From an ecological dynamics perspective, synergies express relationships between their components, namely, cooperation among components' contextual roles to achieve a task goal (see [50], for a detailed discussion). Recently Cabe explored the hypothesis that in fact all (biological) perception engages in the tensegrity-based haptic medium. All movement adjustments involved in active perception affect the organismic tensegrity system [51]. Thus, tensegrity properties enable the synergies underlying complex human movement in task- and context-specific scenarios.

### Biotensegrity is Based on Perception-Action Coupling

Biotensegrity is a functional concept, rather than an anatomical property [52], which implies perception-action coupling, or more generally, sensing-actuating links [36]. Perception-action coupling is situated at the level of the individual-environment system [53, 54]*.* Perception and action regulate goal-directed actions in a given environment, which are adaptive behaviors. A performer is coupled to the environment through informational variables (optics, acoustics, and haptics) but also through the changes in the environment caused by their own actions [51, 52]. Tensegrity structures may be effectors of action (e.g., muscular-ligament-skeletal system) [55], a medium for haptic perception [39], or an organizational structure (e.g., lens of the eyes, [56]). Moreover, individuals are neurobiological degenerate systems, i.e., they can (structurally) vary motor behavior to achieve the same function [57]. In all human action (even at rest), environmental influences (forces and information; in the ecological sense, information is ambient energy distributions, as it happens with light) are omnipresent. Perceiving as it happens in looking, listening, smelling, tasting, touching and, in fact, all exploration of stimulation arrays involve active movement and therefore have impact in the tensegrity structure (see [51] for a detailed discussion). Consequently, the tensegrity system is always in use, and the structure is continuously changing to adapt.

To date, research addressing the relationship between distinct tensegrity structures focused on an intrapersonal approach that assumes there is a physical connection between unit elements. However, in larger systems such as sports teams, the individual components (players) can also be connected by information [53]. The detected information constrains the individual’s behavior in the same way as within-body mechanical forces constrain movement. Moreover, interpersonal movement coordination follows the same self-organizing dynamics [58] as bimanual coordination in an individual [59]. A similar phenomenon was found in individuals acting in coordination to perform a simple [60] or a complex task, such as a football match [13]. These examples highlight how information can connect components in a system similarly to mechanical linkages.

### Tensegrity Properties and Sports Teams

Contemplating the multifractality of tensegrity systems in individual human movement [39], we hypothesized that tensegrity properties can also be expressed in the collective behavior of a group of individuals with common goals (e.g., a sport team). Therefore, how can tensegrity systems properties be related to a sports team’s collective behavior?

To address the property of pre-stress, which is a state of intrinsic tension allowing fast responses to changes in stress anywhere in the system, the question of “what constitutes the intrinsic tension of a sports team?” is of utmost importance. Intrinsic tension is created by past experience, the team sport skill learning, the common path that characterizes a given team when they arrive at a performance context, and the learned and practiced processes, including acting and perceiving affordances of others and for others [7]. Also, more permanent environmental constraints [61], such as rules or court dimensions, influence intrinsic tension. All of these constraints confer information, omnipresent within the system formed by a sport team. Importantly, if when we consider a tensegrity in a physical structure, physical tension is means by which all the elements are linked; in this case it is informational tension that links the elements (players). However, how does a team maintain its intrinsic informatinal tension given the dynamic nature of the task? The players adjust their actions to the information available in every moment, which means that they change over time the structure they form, and thus they change the team’s informational tension. The challenge is to keep the informational tension in a dynamic state that provides structure (team) stability and ensures responsiveness.

The property of energetic efficiency indicates that configurations that favor efficiency and are capable of storing energy within the system rely on the team’s ability to express adaptive behavior. A sports team expressing adaptive behavior exhibits flexibility and variability to respond to events at any time. Flexibility to adapt facilitates the efficiency of the structure (the team) in response to the adversaries’ actions, in particular, and game dynamic constraints, in general. A loss of efficiency in the structure can be linked to more uncoordinated actions such as unnecessary redundancies (e.g., players invading other players’ areas of responsibility) or detrimental delays (e.g., players not positioning favorably to perform his or her share or to compensate teammates’ less successful actions). For example, experienced soccer players are more efficient (fewer positioning corrections) than less experienced players [62] and are more prompt to develop coordination tendencies in soccer tasks [63].

Tensegrity structures exhibit nonlinear viscoelastic type properties such as non-linear stiffening, which allows tensegrity systems to become stronger when subjected to higher forces. A sports team pressed by higher tension (e.g., expert adversaries and higher game intensity) needs to keep the structure stable to maintain adaptive behavior under such constraints. There is evidence that sports teams, which exhibit stability and efficiency in their coordinated actions, can overcome constraints that are theoretically inhibitory of success [64].

The property of omnidirectional stability, which is the ability to maintain functional properties independently of gravity direction, is related to sports teams in terms of space. Synchronization among players is not necessarily an indication of adaptive behavior. To be relevant, synchronization among players needs to harness local constraints, namely, the space where it occurs [12]. Research on this topic addresses mainly longitudinal and lateral coordination [13, 65, 66]. However, different team sports have different specific constraints. For example, for volleyball, team structure can be defined in three dimensions, including height. Only by presenting the properties listed above a sports team can exhibit the structural stability and adaptability of a tensegrity system. The question is how can this be captured?

## Geometrical Configurations and Architectural Control

### Form-Finding in a Team: a Quest for Structural Stability

Sports teams adopt positional or geometrical configurations in the field [5] to cope with the demands of the match and facilitate point-scoring while simultaneously preventing the opponent team from scoring [67]. Sports teams try to maintain structural stability to improve performance [68]. However, while geometrical configurations impose team constraints regarding the positions of players and their priority links, they also need to be adaptable to match dynamics (i.e., the evolving of match events) [9]. Research in interpersonal coordination has been conducted in different collective [65, 66, 69] and individual sports [70–72] and at different levels of social complexity (i.e., dyads, sub-groups, teams) [73]. It is clear from this body of research that the specific constraints of each sport, levels of social complexity, or sub-phases of the same sport (e.g., attacking, defending) are associated with different patterns of coordination. In sports teams, intra- and inter-team co-adaptation and coordination tendencies vary among sports and within the same sport. Even when both teams of the same sport adopt similar positional distributions (e.g., 4:3:3 in soccer), they express different degrees of efficiency in their collective actions [74], indicating that the main feature of team performance is the dynamic capacity for maintaining responsive actions to local constraints. Geometrical configuration dependency between teams is eventually more evident in invasion sports where teams share the same space [22]. In net sports, since teams cannot recover the ball from the adversary space, the dependency of the positional configuration might be less dynamic, and previously trained plays may become more resistant to perturbation.

**Fig. 1:**
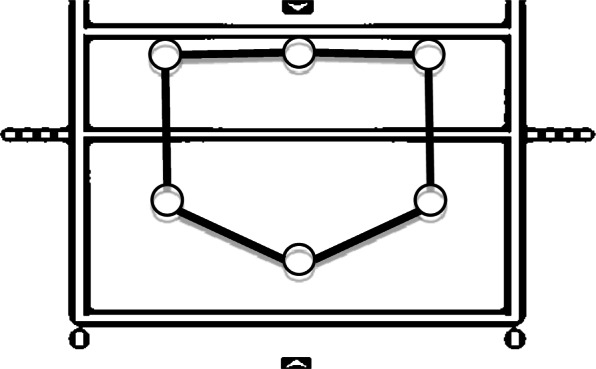
Typical defensive geometrical distribution of players in a volleyball team. The figure represents half of the volleyball court, where the top is the net and the bottom and right and left lines are the marked limits of the pitch. The six circles represent the players of the team defending this part of the pitch. The line parallel to the net is the 3-m line, which delimits the zone for the attackers

Given the behavior of mechanical tensegrity structures, which tend to maintain stability and integrity under external forces [75], stable geometrical configurations (i.e., adequate positional occupation to proficiently adapt to game dynamics) [76] similar to tensegrity structures emerge during a match. For example, the positions of volleyball players before initiating their actions to defend an opponent’s attack for the following geometrical configuration (Fig. 1).

A typical defensive shape is maintained between matches and volleyball teams [77] because it offers an effective configuration for adapting efficiently and rapidly to the adversary’s actions. Similarly, engineering tensegrity structures “[…] provide the potential to control their shape and adapting to changing tasks and environments […] these systems exhibit geometrically nonlinear behavior and are strongly coupled […]” ([36], p. 1454–1455). Determining a stable geometrical configuration in a tensegrity structure is referred as the “form-finding” problem, and it must consider (i) the patterns of connectivity that enable a stable state or tensegrity and (ii) the length parameters of rigid and tensile elements for a given stable connectivity pattern [78]. Form finding and structural stability also occur in team sports; for example, in football, skilled players tend to be distributed by design and become tactically balanced. Designs for space occupation that form a geometrical shape maintain the distances between elements within certain parameters and promote team performance [22, 76, 79]. Adaptive behaviors to maintain connections with teammates during a match are more robust in skilled players [22, 80]. Several methods are currently available for form-finding, including non-linear programming, dynamic relaxation, and calculation of force density. These approaches calculate parameter values [81] and/or connectivity patterns (e.g., genetic algorithm) [78], and they can serve as an inspiration to team sports performance analysis. Thus, the hypothesis presented here offers a new avenue to explore the tensegrity properties or form-finding dynamics in team sports performance.

### Control: Architectural-Constrained Solutions

In the human body, baseline levels of pre-stress or preexisting tension, ensure a constant balance between internal and external forces. Postural states are associated with changes in internal forces, while external forces influence postural transitions [82]. Tensegrity structures adapt to the environment by changing intrinsic stress with sensors and actuators [37]. Considering a sports team as a group-tensegrity structure, the players’ perception and action processes allow the tensegrity structure to emerge. While team actions can be highly plastic and dependent on immediate constraints (e.g., structure complexity of attack coverage in volleyball is dependent on attack tempo) [83], they may also benefit from and usually rely on strategy or design based on player spatial distribution [84]. For example, it is common for players to have so-called “areas of responsibility” in defending or attacking sub-phases of the game [85].

In sports teams, geometrical configurations must allow fluid sharing of information between players who move freely but not separately from each other to search for efficient solutions. From the group-tensegrity hypothesis we are presenting, external constraints acting on the structure are mainly informational and omnipresent over time. Therefore, stability must be dynamic. In a weak tensegrity team’s organization, its geometrical configuration, stable at one point in time, might lose stability as context unfolds. Only geometrical team configurations capable of sustaining tensegrity-like properties will ensure adaptive dynamic stability. Several models for the dynamic control of tensegrity structures [36, 86] offer insights for the analysis of sports team behavior. “Deployment” is the process whereby a mechanical tensegrity structure in equilibrium changes to another state [87], such that a deployment path can be predicted within an equilibrium manifold. Can a deployment path favoring adaptive processes at different time scales be predicted for a sports team? Although equilibrium manifold and control variables can be calculated in tensegrity structures [87], this is not applicable (it is unrealistic to create a space state of all possible configurations) in sports teams. However, control variables such as length of *hard* and *soft* components may eventually be found in tensegrity structures connected by information, for example, in the coupling strength of players’ shared actions and perceptions. Considering a group-tensegrity system connected by information, candidates to control variables would be available time to perceive (e.g., by changing ball or opponents’ speed), social density (e.g., by shifting the numerical ratio between teams), or ball proximity to target areas (e.g., changing the distance to the goal in soccer or to the net in volleyball). These variables will test the system in its stability and responsive capability, eventually leading to differences in players’ phase and distance relations. Importantly, McGarry et al. [88] argue that sports teams may or may not exhibit high variability before the transition from a stable state to another [88].

Biotensegrity systems tend to be more complex than mechanical systems. However, at the cellular level, determining factors that produce ordered system-behavior have been identified [38]. Thus, models of larger tensegrity systems, such as sports teams, can be conceived.

## Sports Team as a Group Tensegrity System: an Exploration in Volleyball

**Fig. 2:**
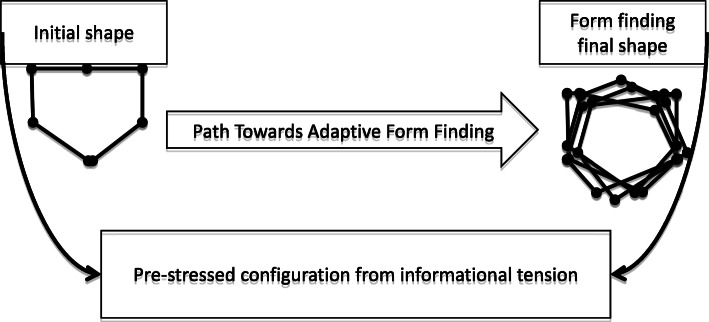
Model of a sports team as a group-tensegrity system. Example of a volleyball team in defensive tasks, adaptive form-finding from pre-stressed configurations

A volleyball team in defensive tasks can be conceived as a group-tensegrity system (Fig. 2) with essential pre-stress and energy efficiency properties eventually related to a controlled path towards an adaptive form-finding. As such, in a volleyball match, the players are connected informationally (e.g., via visual perception), and pre-stress as a pre-existing condition results from pre-defined strategies and learned shared-affordances. The most crucial pre-stress part of the performance is a result of learning from practice. Indeed, the players can practice to become perceptually attuned and calibrated to the shared affordances of others and for others in their team [7]. This process of learning shared-affordances is enhanced when practice offers environmental relevant properties mimicking the match situation and which are therefore representative of performance environments [73, 89]. Behavior organization unfolds during the play and is supported by movement and on-line information detection [90] but constrained by the structure’s pre-stress (Fig. 2).

**Fig. 3:**
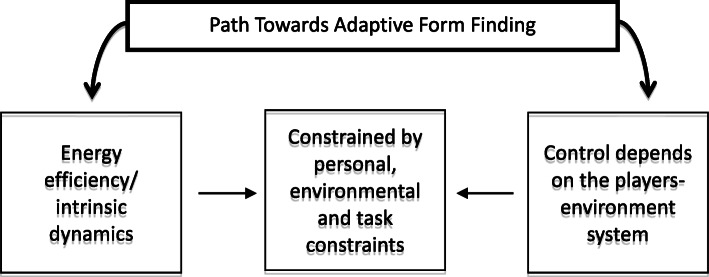
Model of a sports team as a group-tensegrity system. Example of a volleyball team in defensive tasks, in which adaptive form-finding control and intrinsic dynamics are constrained by personal, environmental, and task constraints

While the opponent team develops their offensive play, a path to form-finding is initiated but hardly pre-determined, as athletes reorganize movements according to available informational constraints [91]. There is a close relationship between task control (i.e., adaptive behavior) and energy efficiency (i.e., intrinsic dynamics of each player), whereby higher expertise is linked to more efficient cooperation among players [62, 63, 92]. Energy efficiency in adaptive behavior should not be understood in absolute terms (less energy) but instead in the adequate movement variability/adjustment to meet task demands [93]. The control of system behaviors depends on functional variability (e.g., by exploring, selecting, or abandoning organizational states) [94–96] to manifest flexibility and self-organization [97]. Thus, we suggest that such properties enhance the possibilities of discovering stable geometrical configurations and, by extension, the chances of success in defensive play are increased (Fig. 3).

An experimental test for investigating tensegrity principles in a volleyball team may be achieved by comparing a set of spatiotemporal (e.g., players phase and distance relations) variables between successful and unsuccessful defensive plays. Context-dependent collective behavior has been previously measured in sports teams [12, 13], and such methods may be useful to capture pre-stress in the system (volleyball team in defensive tasks) during the path towards adaptive form-finding. Finally, movement variability dynamics [98, 99] and the dynamics of space occupation can both contribute to determining how intrinsic dynamics and geometrical configurations evolve to adapt to ongoing constraints [79].

## Conclusion

Research-based on ecological dynamics methods [5] has previously described synergic behavior in team sports. Here, we propose that a new approach based on the concept of tensegrity may raise new questions and accurately measure team sports dynamics and organization, thereby potentially offering valuable novel insights. In biological systems physically connected, tensegrity principles can be observed from a nanoscopic [42] to a macroscopic scale [39, 48]. We propose that systems connected informationally as a group-tensegrity structure, such as sports teams, may follow a similar set of principles to achieve synergic adaptive behavior. Given that structure and function are highly complementary [100], we hold that group-tensegrity may inform in a structure to function direction (initial conditions and team geometrical configurations over time), whereas team synergies inform in a function to structure sense (dimensional compression, reciprocal compensation, interpersonal linkages and degeneracy), being, thus, complementary approaches within ecological dynamics.

The group-tensegrity hypothesis is a path that is opened to guide future (and needed) research. However, such research needs to consider the specific constraints of each sport, and the kinds of informational variables that challenge the properties of the system and the adjustments it reflects. By knowing these properties of team function and structure dynamics, training methods can be tested, and their efficacy monitored over preparatory cycles.

## Data Availability

Not applicable.
